# Interaction between *Rag* genes results in a unique synergistic transcriptional response that enhances soybean resistance to soybean aphids

**DOI:** 10.1186/s12864-021-08147-3

**Published:** 2021-12-11

**Authors:** Martha I. Natukunda, Jessica D. Hohenstein, Chantal E. McCabe, Michelle A. Graham, Yunhui Qi, Asheesh K. Singh, Gustavo C. MacIntosh

**Affiliations:** 1 Roy J. Carver Department of Biochemistry, Biophysics and Molecular Biology, Iowa State University, Ames, IA 50011 USA; 2 Corn Insects and Crop Genetics Research, USDA-ARS, Ames, IA 50011 USA; 3 Department of Agronomy, Iowa State University, Ames, IA 50011 USA; 4 Department of Statistics, Iowa State University, Ames, IA 50011 USA

**Keywords:** Gene pyramiding, Soybean, Soybean aphids, Aphid resistance, *Rag* genes, RNA sequencing, Synergistic effect

## Abstract

**Background:**

Pyramiding different resistance genes into one plant genotype confers enhanced resistance at the phenotypic level, but the molecular mechanisms underlying this effect are not well-understood. In soybean, aphid resistance is conferred by *Rag* genes. We compared the transcriptional response of four soybean genotypes to aphid feeding to assess how the combination of *Rag* genes enhanced the soybean resistance to aphid infestation.

**Results:**

A strong synergistic interaction between *Rag1* and *Rag2*, defined as genes differentially expressed only in the pyramid genotype, was identified. This synergistic effect in the *Rag1/2* phenotype was very evident early (6 h after infestation) and involved unique biological processes. However, the response of susceptible and resistant genotypes had a large overlap 12 h after aphid infestation. Transcription factor (TF) analyses identified a network of interacting TF that potentially integrates signaling from *Rag1* and *Rag2* to produce the unique *Rag1/2* response. Pyramiding resulted in rapid induction of phytochemicals production and deposition of lignin to strengthen the secondary cell wall, while repressing photosynthesis. We also identified *Glyma.07G063700* as a novel, strong candidate for the *Rag1* gene.

**Conclusions:**

The synergistic interaction between *Rag1* and *Rag2* in the *Rag1/2* genotype can explain its enhanced resistance phenotype. Understanding molecular mechanisms that support enhanced resistance in pyramid genotypes could facilitate more directed approaches for crop improvement.

**Supplementary Information:**

The online version contains supplementary material available at 10.1186/s12864-021-08147-3.

## Background

Plants are in a constant battle against pathogens and pests. A common outcome of the selective pressure imposed on plants by pests is the evolution of plant resistance genes (*R*) that can recognize pest challenges [1]. The detection triggers a physiological response that reduces the invader’s ability to colonize or feed on the plant. In turn, the selective pressure thus imposed on the pest can result in the evolution of different strains or biotypes with the ability to evade recognition by specific *R* gene products. This well-defined evolutionary arms race has been identified as one of the processes that shape plant and pest evolution [2]. Many types of *R* genes have been identified [1], although the most abundant and well-characterized belong to the intracellular nucleotide-binding/leucine-rich-repeat (NLR) class [3]. These receptors detect pathogen or herbivore effectors and evoke a defense response, effector-triggered immunity (ETI) that shares common signaling events and outputs with the basal defense response triggered by recognition of pathogen or herbivore associated molecular patterns (PTI); although the ETI response is faster and seems to overcome negative regulators that normally temper the strength of the PTI response [3].

Plant response to stress needs to display both robustness and plasticity simultaneously [4]. In the case of plant immunity, robustness is necessary to mount a strong defense in the presence of a large variety of pests, while plasticity is needed to display a different flavor of the defense arsenal as specific pathogens change [3, 5]. Activation of ETI can be triggered by direct interaction between an effector and a NLR, indirectly through NLRs that can sense host targets modified by effectors, or by sensing effector-decoy interactions [6]. It has been proposed that, upon activation, sensor NLRs transmit the signal to helper NLRs that do not interact directly with effectors [5, 6]. Moreover, while a large number of sensor NLRs exist in many angiosperm species, helper NLRs are fewer; thus *R* gene signaling converge on helper NLR nodes to create a regulatory network with built-in redundancy [5, 7]. These regulatory networks also converge downstream in a series of phytohormone crosstalk events, mainly synergistic or compensatory interactions between the jasmonate (JA) and salicylate (SA) pathways, that contribute to the connectivity of the network [8, 9]. Finally, these signals trigger a transcriptional reprogramming that is used by the plant to mount different chemical, structural, and physiological changes depending on the nature of the attacking pest [3]. Signal integration that leads to a specific transcriptional response is controlled by transcription factor (TF) regulatory networks that rely on a selected group of TFs and interacting proteins to provide specificity [10, 11]. This network architecture with highly connected nodes, including helper NLRs, phytohormone regulatory factors, and TF hubs, imparts plasticity and robustness to the system.

*R* genes are commonly used as part of management strategies to control pests in crop production, as a sustainable alternative to chemical treatments. However, the selective pressure exerted on the pathogens or insects favors the evolution of pests, negatively impacting the durability of individual *R* traits [12, 13]. One strategy used by plant breeders to overcome this limitation is the use of gene pyramids: the incorporation of multiple different *R* genes able to control a specific pathogen or insect in an individual crop variety [14]. Gene pyramids have been successfully used to manage different bacterial, fungal, and insect pests in a variety of species including rice, wheat, and soybean, among others [14, 15]. In many cases, these crop varieties with multigenic resistance have enhanced pest control when compared with monogenic lines, indicating an additive interaction between *R* genes [14–17]. In other cases, however, this interaction may result in incompatibility and less favorable outcomes [18]. These *R* gene interactions suggest that each *R* gene triggers a subset of the defense arsenal. The combination of multiple *R* genes results in complementary, or antagonistic, outcomes that can produce plants with a more robust or compromised defense response depending on the nature of this interaction [15]. While these interactions are well-described from a phenotypic perspective and pyramids are used frequently in breeding programs, the molecular events underlying the observed traits are normally not known.

The soybean (*Glycine max*)-soybean aphid (*Aphis glycines*) system is an attractive model to study the effects of gene pyramiding. The soybean aphid, an invasive insect species, is one of the most economically damaging pests of soybeans in the United States, causing up to 40% yield reduction if left unmanaged [19, 20]. Resistance to soybean aphids is provided by *Resistance to* *Aphis* *glycines* (*Rag*) genes. Twelve *Rag* genes and four aphid resistance quantitative trait loci have been identified to date [21], although none have been cloned so far. The resistance provided by *Rag1* and *Rag2* has been well-characterized, and transcriptome studies describing the response of plants carrying either gene to soybean aphids are available [22–24]. The *Rag1* gene was the first resistance gene to be identified in the soybean cultivars Dowling and Jackson [25–27]. The gene is located in a 115 kb interval on chromosome 7 [28]. *Rag2* was identified in two plant introductions, PI 243540 and PI 200538 [29, 30], and it is located in a 54 kb interval on chromosome 13 [31]. Both these regions contain NLR genes which have been proposed as candidates for the resistance genes [28, 31]. Both the *Rag1* and *Rag2* genes have been shown to primarily confer an antibiosis type of aphid resistance [32]. The *Rag1* transcriptional response to aphid feeding involved repression of transcripts related to cell wall modifications, induction of SA regulated plant defense, induction of the phenylpropanoid pathway, and upregulation of cuticle production [22, 23]. The *Rag2* transcriptional response to aphid feeding involved upregulation of genes involved in cell wall modifications, secondary metabolism, hormone metabolism and stress signaling while transcripts involved in carbon metabolism and photosynthesis were downregulated [24].

While different *Rag* genes are available for breeding programs, the discovery of soybean aphid biotypes [33–35] that can successfully colonize resistant soybean genotypes led to the development of soybean lines carrying gene pyramids with two or three resistance genes [17, 36–38]. Greenhouse and field phenotypic studies showed that pyramid lines carrying *Rag1* and *Rag2* can control aphid populations more effectively than monogenic resistance lines [17, 36, 39], and no adverse effects have been reported for this interaction. Given that each *Rag* gene seems to trigger a somewhat different transcriptional response, we hypothesized that the enhanced resistance observed at the phenotype level would be based on an additive effect at the transcriptional level, that is, the transcriptional response of the *Rag1/2* pyramid should overlap with the transcriptional responses of *Rag1* plus *Rag2*. Alternatively, the interaction of signaling events triggered by *Rag1* and *Rag2* in the pyramid could lead to an output different from the addition of individual *Rag* gene responses. In this case, the interaction can be considered synergistic and is measured at the molecular level by the number of genes whose expression changes significantly only in the pyramid. This type of transcriptional synergistic response has been previously observed in the cellular response to drug combinations when compared to the response to monotherapies [40]. The enhanced transcriptional response, due to either additive or synergistic interaction, would then translate into a more robust resistant phenotype.

To test these hypotheses, we conducted a transcriptome analysis using RNA sequencing (RNA-seq) to determine the molecular response of four soybean genotypes to soybean aphids 6 or 12 h after infestation. The soybean genotypes used were: IA3027RA12 which contains both the *Rag1* and *Rag2* genes, IA3027RA1 which has *Rag1* alone, IA3027RA2 which has *Rag2* alone and IA3027, the susceptible control. These soybean genotypes are referred to as *Rag1/2*, *Rag1*, *Rag2* and susceptible, respectively. Our results showed that, unexpectedly, the *Rag1/2* response presents a unique transcriptome signature that indicates a synergistic interaction between the *Rag1* and *Rag2* genes.

## Results

### Confirmation of enhanced resistance in the pyramid genotype

**Fig. 1:**
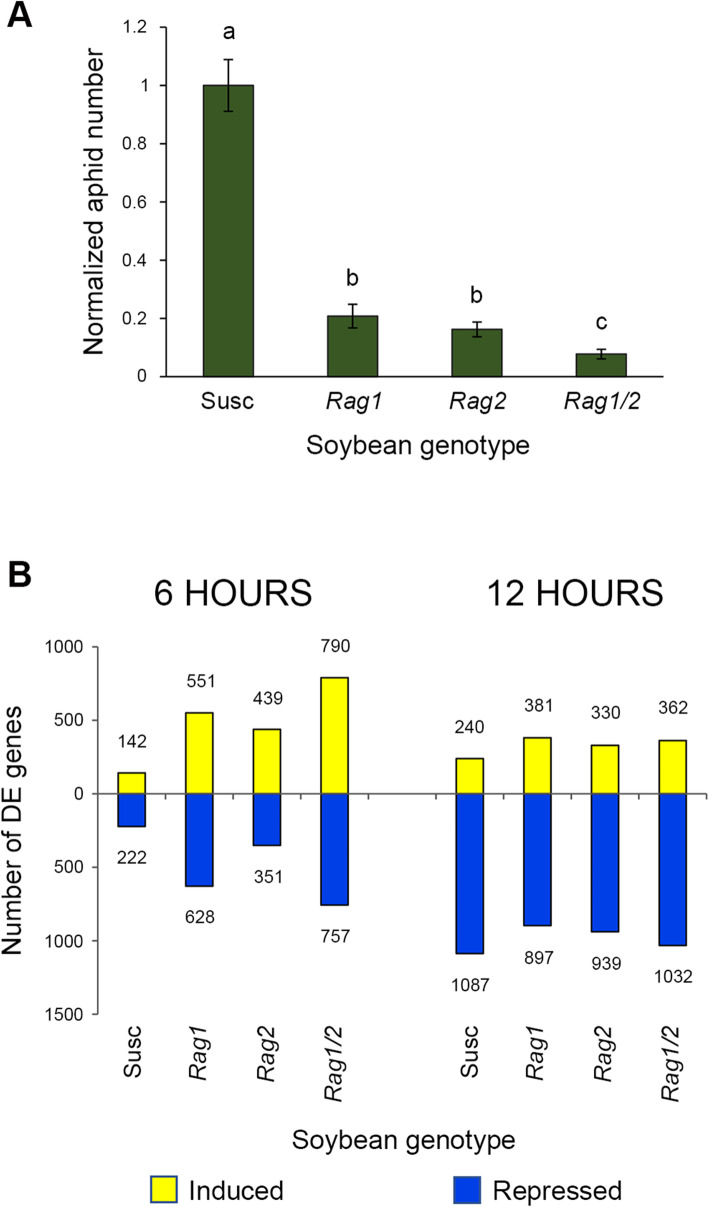
***A****. aphid* resistance phenotypes of the genotypes used in this study. Phenotyping results for the four soybean genotypes obtained using no-choice experiments conducted in growth chambers. Data was normalized to the susceptible control, IA3027. Statistical data analysis for each comparison was done using Student’s *t*-tests (Two-Sample Assuming Unequal Variances). Error bars represent the standard error. Different letters indicate statistically significant differences (*p* < 0.05). ***B***. Global changes in the soybean transcriptional response to aphids. Total number of DE genes for aphid versus mock comparisons for each soybean genotype at each time point is shown as induced genes (positive fold change) and repressed genes (negative fold change). Total number of induced and repressed genes for each comparison are indicated next to each of the bars

A no-choice experiment was used to determine whether the resistance phenotype of the monogenic and pyramid genotypes could be observed using our experimental conditions. As expected, the *Rag1/2* soybean genotype had significantly reduced aphid populations compared to genotypes with the *Rag1* or *Rag2* gene alone, both of which had significantly fewer aphids than the susceptible control (Fig. 1A). There were no significant differences in aphid populations between soybean genotypes that contained the *Rag1* or *Rag2* gene alone.

### Transcriptional response to soybean aphids

The transcriptional response to aphids of each genotype was assessed to understand the molecular effect of pyramiding aphid resistance genes in soybean. The response was analyzed at 6 and 12 h after infestation because previous experiments have shown that the most significant transcriptome changes in resistant plants are observed earlier than 24 h after aphid infestation [22–24, 41].

In total, 1,042,480,636 reads were generated from 48 leaf samples, with an overall mapping rate of > 95% with reference to version 2 of the Williams 82 soybean genome [42]. Of those, 4646 differentially expressed (DE) genes responded to soybean aphids in one or more of the four soybean genotypes for the eight (aphid versus mock) comparisons made for both time points (Additional File 1). The number of DE genes for each of the three aphid-resistant soybean genotypes was larger than in the susceptible response 6 h after infestation. In contrast, at the 12 h time point, the number of DE genes was similar among the four soybean genotypes. The majority of the DE genes (> 70%) were repressed for all four soybean genotypes (Fig. 1B) at the 12 h time point.

**Fig. 2:**
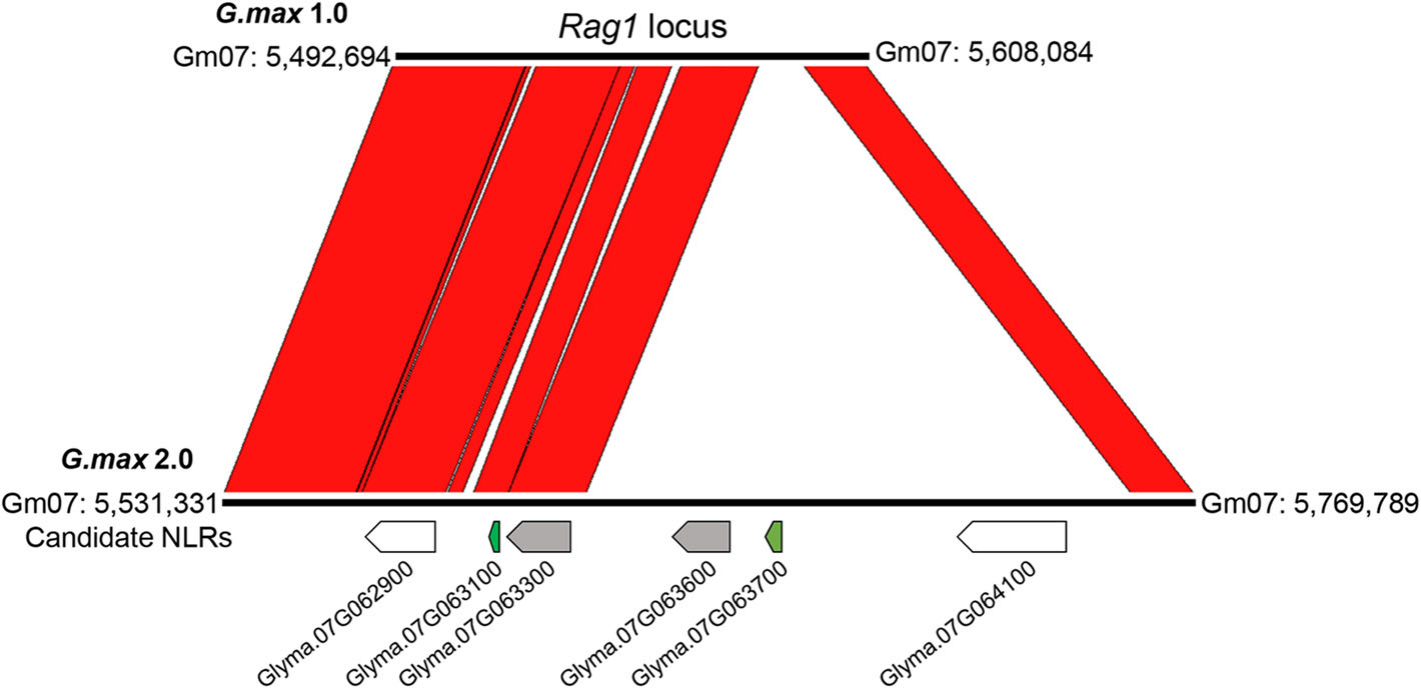
Comparison of the *Rag1* locus in versions 1 and 2 of the Williams 82 genome. The top line represents the sequence of the *Rag1* locus in version 1 of the soybean genome as reported by Kim et al. (2010). The bottom line represents the *Rag1* locus in version 2 of the Williams 82 genome assembly. Conserved regions are represented by red shading. Candidate NLRs are represented as arrows and color-coded as follows: not differentially expressed (white), expressed more in the susceptible genotype (grey) or expressed more in the resistant genotypes (green)

We also examined the expression differences between soybean genotypes in the absence of aphids by comparing mock samples across genotypes and time points (Additional File 2). Comparing the three resistant genotypes to the susceptible line, and the *Rag1/2* pyramid resistance line to the single resistance gene genotypes, at six and twelve hours, identified between 75 and 218 DE genes per comparison. Among these genes, of specific interest were those located within the *Rag1* [28] and *Rag2* loci [24]. In version 2 of the soybean genome, the *Rag1* locus corresponds to Gm07: 5,531,331 to 5,769,789 and contains the range of genes from *Glyma.07G062300* to *Glyma.07G064700* (Fig. 2). Six genes from this region were DE between genotypes when comparing *Rag1* to susceptible, *Rag1/2* to susceptible or *Rag1/2* to *Rag2* (*Glyma.07G062400*, *Glyma.07G063100*, *Glyma.07G063300*, *Glyma.07G063600*, *Glyma.07G063700* and *Glyma.07G064400*, Table 1). Four of the DE genes in the region have BLASTP homology to soybean NLRs (E = 0, *Glyma.07G063100*, *Glyma.07G063300*, *Glyma.07G063600* and *Glyma.07G063700*). Of these, *Glyma.07G063100* was expressed at somewhat higher level in *Rag1*-containing genotypes in three comparisons (Table 1), while *Glyma.07G063700* was expressed at much higher level in all *Rag1*-containing genotypes across all six comparisons (FC between 5.6–10.4; Table 1). This suggests that the orthologs of *Glyma.07G063700*, or less likely *Glyma.07G063100*, correspond to *Rag1*. These genes were not identified as candidates for *Rag1* by Kim et al. [28], who used the first release of the soybean genome (*G. max* 1.0) in their study. *Glyma07g06910* (corresponding to *Glyma.07G063100*) was predicted as a low confidence gene model with no NLR homology in the first genome release. The *G.max* 1.0 gene corresponding to *Glyma.07G063700* (*Glyma7G07145*) was located outside of the genomic interval corresponding to *Rag1.* However, between the *G. max* 1.0 and *G. max* 2.0 versions of the soybean genome, additional sequencing and markers were used to fill gaps and improve sequence assembly. The genomic interval between the markers used to map *Rag1* in genome version 2 has increased by 123 kb and now includes *Glyma.07G063700*, making it a high priority candidate for *Rag1* (Fig. 2).

**Table 1:** Differentially expressed genes among genotypes, in the absence of aphid feeding near the *Rag1* and *Rag2* loci

Locus	Gene ID	Comparison					
***Rag1***							
		6 h			12 h		
		*Rag1*vSusc	*Rag1/2*vSusc	*Rag1/2*v*Rag2*	*Rag1*vSusc	*Rag1/2*vSusc	*Rag1/2*v*Rag2*
	*Glyma.07G064400*	n.s.	1.25139	n.s.	0.90248	0.92288	n.s.
	Glyma.07G063700*	2.48920	3.22033	3.37710	3.01142	3.13451	3.24709
	Glyma.07G063600*	n.s.	-1.97796	-1.97610	-1.84461	-1.70881	-1.85213
	Glyma.07G063300*	-0.92302	-1.40440	-1.28773	-1.29225	-1.14970	-1.24997
	Glyma.07G063100*	n.s.	n.s.	n.s.	0.57516	0.68902	0.72726
	*Glyma.07G062400*	n.s.	n.s.	n.s.	n.s.	n.s.	-0.62122
***Rag2***							
		6 h			12 h		
		*Rag2*vSusc	*Rag1/2*vSusc	*Rag1/2*v*Rag1*	*Rag2*vSusc	*Rag1/2*vSusc	*Rag1/2*v*Rag1*
	*Glyma.13G190900*	-1.33233	-1.35883	-1.44740	-1.70851	-1.75952	-1.49086
	Glyma.13G190800*	-1.85926	-1.95789	-1.81795	-1.84813	-1.62494	-1.57998
	Glyma.13G190400*	-1.70636	-1.87610	-1.56779	-2.00623	-1.80790	-1.67855

Comparisons between genotypes with or without a specific *Rag* gene were carried out, as indicated, and LogFC is presented for DE genes near the *Rag1* or *Rag2* loci. The genes underlined correspond to candidate *Rag* genes based on fine mapping [28, 31]. The gene in **bold** indicates a new candidate identified by our analysis. Genes with homology to NLRs are indicated by *. “n.s.” indicates that difference in expression for the gene was not statistically significant in the comparison

The same approach was used to identify candidate genes for *Rag2.* In *G. max* 2.0, the *Rag2* locus corresponds to Gm13: 30,412,470 to 30,466,615 and contains the range of genes from *Glyma.13G190400* to *Glyma.13G191200*. Three genes from this region were DE between genotypes when comparing *Rag2* to susceptible, *Rag1/2* to susceptible or *Rag1/2* to *Rag1* (*Glyma.13G190400*, *Glyma.13G190800* and *Glyma.13G190900*, Table 1). Of these three, two have BLASTP homology to soybean NLRs (E = 0, *Glyma.13G190400* and *Glyma.13G190800*), however, both were repressed across all six genotype comparisons. Unexpectedly, both genes were repressed in response to aphids in *Rag1* at 12 h (Additional File 1) suggesting the *Rag1* and *Rag2* loci could somehow interact. *Glyma.13G190400* (*Glyma13g25970* in *G. max* 1.0) was proposed as a candidate for *Rag2* gene based on gene expression [24], while *Glyma.13G190800* (*Glyma13g26000* in *G. max* 1.0) has been proposed as the candidate *Rag2* gene based on fine mapping [31]. Repression of these genes in our experiment could suggest they are not candidates for *Rag2*, but it is important to note that the time points used in this study are just a small snapshot of the resistance response. It has previously been proposed that the presence of *Rag1* and *Rag2* genes causes constitutive expression of some defense genes even in the absence of herbivores [23, 43]; however, this effect seems to be minor for the genotypes analyzed here.

### Transcriptional effect of pyramiding *Rag1* and *Rag2* genes at the global level

**Fig. 3:**
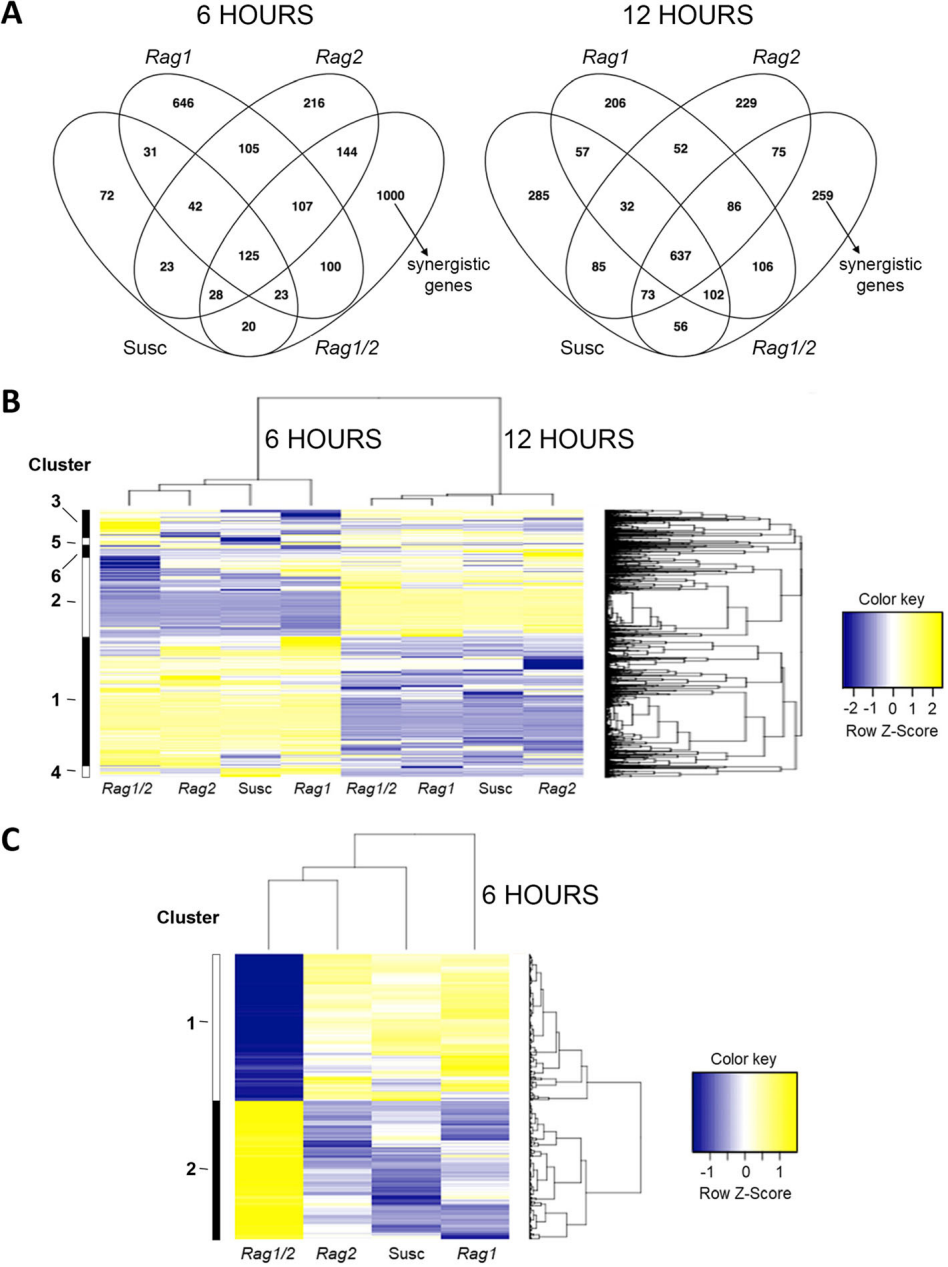
The pyramid genotype has a unique transcriptome response. **A**. DE genes in each genotype after aphid infestation for 6 h (left) and 12 h (right) unique to each genotype or shared among different genotypes. The DE genes unique to the *Rag1/2* response were designated as the “synergistic genes” set. **B**. Clustering heatmap showing expression patterns for the 4646 genes differentially expressed after aphid infestation in any genotype after 6 h or 12 h. Each column represents the aphid versus mock comparison for each soybean genotype and time point combination. Each row represents a gene. Genes included in this figure are differentially expressed for at least one comparison. Z-scores were calculated for each gene in all comparisons. Soybean genotypes are designated as: Sus: Susceptible, R1: *Rag1*, R2: *Rag2,* and R1R2: *Rag1/2*. **C**. Heatmap corresponding to the 1000 genes differentially expressed exclusively in the pyramid genotype at 6 h after aphid infestation, referred in the text as the synergistic response

We hypothesized that the *Rag1/2* pyramid genotype response to aphids would be the results of an additive effect of the monogenic genotypes, i.e. the DE transcriptome of the *Rag1/2* plants would be the sum of DE genes in the *Rag1* response and the *Rag2* response. In this case, we expected a nearly complete overlap such that there would be few DE genes unique to the *Rag1/2* transcriptome response to soybean aphids. Alternatively, a synergistic response would be indicated by a set of genes only DE in the pyramid and absent from either individual *Rag* genotype response. When the lists of DE genes for each genotype were compared, there were unique and common gene sets at each time point (Fig. 3A). *Rag1/2* showed large unique DE sets at both time points (1000 genes at 6 h and 259 genes at 12 h), indicating that pyramiding the *Rag1* and *Rag2* genes in one soybean genotype results in a synergistic effect on aphid resistance at the transcriptome level. The DE genes unique to the *Rag1/2* response are referred to as “synergistic genes.” The *Rag1/2* synergistic response to soybean aphids comprised 65% of the DE genes at 6 h and 19% of the DE genes at 12 h. There was a fivefold increase in the number of DE genes that were common to all four soybean genotypes at 12 h when compared to the 6 h time point. The number of other unique and common DE genes varied among respective comparisons (Fig. 3).

In total, there were 4646 aphid-responsive genes in all aphid versus mock comparisons at both time points. Cluster analysis was conducted on these genes to visualize how aphids impacted gene expression on a global level. The DE genes modified by aphids clustered by time point (Fig. 3B). Interestingly, at 6 h, the *Rag1/2* gene expression pattern was more similar to that of *Rag2*, while at the 12 h time point the *Rag1/2* response was closer to the *Rag1* response. Based on similarity in gene expression pattern, DE genes grouped into six clusters (Fig. 3B). Expression patterns for DE genes on the clustering heatmap showed a characteristic set of genes that were induced by aphids at 6 h in all soybean genotypes but repressed at 12 h (clusters 1 and 4). Other sets of genes were mostly repressed at 6 h but induced 12 h after aphid infestation (clusters 2 and 5, Fig. 3B). Subsets located in clusters 2 and 3 with distinctive expression patterns in the 6 h *Rag1/2* response contained some of the synergistic genes. The distinctive region in cluster 2 had DE genes that were strongly repressed in the 6 h *Rag1/2* response only but not modified by aphids in the other three soybean genotypes. In cluster 3, the distinctive region contained DE genes that were strongly induced in the *Rag1/2* response only. The rest of the synergistic genes were distributed among the clusters with differences in expression patterns.

Given the size of the early synergistic gene set, this 6 h set (1000 DE genes, Additional File 1) was reanalyzed to evaluate the possibility that these genes were the result of a quantitative additive effect, that is, genes that were marginally regulated in the monogenic genotypes and only became DE in the pyramid as a result of additive effects that placed their expression above our arbitrary FDR cutoff. A cluster analysis of the synergistic genes for the 6 h time point (Fig. 3C) identified two clusters of similar size that were defined by the *Rag1/2* genotype response (either repressed or induced). Of the 1000 *Rag1/2* DE genes depicted, 664 (66%) were significant in *Rag1/2* (FDR < 0.05), but not significant in *Rag1* or in *Rag2* using a relaxed cutoff (FDR > 0.25). A similar approach has been used to differentiate genes affected by combined stresses versus the effect of individual stress conditions [44]. We also compared the observed log2FC values for the 6 h synergistic gene dataset to the expected additive values (log2FC*Rag1* + log2FC*Rag2*) (Additional File 3, Fig. S1) in aggregate using a *χ*^2^ test. This analysis showed that the observed values for the full 6 h synergistic dataset are significantly different from the expected additive values (*χ*^2^ = 2.96^− 28^). Analysis of the induced or repressed gene subsets, however, showed that observed values for repressed synergistic genes are not significantly different from expected additive changes (*χ*^2^ = 1), while observed values for induced synergistic genes are significantly different from expected additive values (*χ*^2^ = 4.08^− 92^). These results indicate that the synergistic gene set is, at least in part, uniquely regulated by aphids when the two resistance genes are present in the same genotype.

### Dynamics of the response to aphids for each genotype

To determine the time progression of the response of each soybean genotype to aphids, gene lists (aphid versus mock) for each soybean genotype at 6 h and 12 h were compared (Additional File 3, Fig. S2). Overall, there was very little overlap of DE genes at both time points (< 6%) within each soybean genotype. The number of DE genes that were common at both time points for each soybean genotype was: 38 for the susceptible, 108 for *Rag1*, 114 for *Rag2*, and 108 for *Rag1/2*. Within each soybean genotype, some DE genes changed in the same direction at both time points, but others showed opposite regulation (induced at 6 h but repressed at the 12 h time point or vice versa). The very small overlap of DE genes between the two time points for each soybean genotype indicates a dynamic response to aphids for all genotypes. Moreover, given the limited overlap in the transcriptional responses at 6 h versus elevated similarity at 12 h, the results suggest that earlier events determine the differences in the resistance phenotype observed for each genotype.

Importantly, most 6 h synergistic genes (910 genes out of the 1000 gene set) are not DE in the 12 h response in *Rag1/2* or any other genotype, suggesting that the 6 h synergistic response in the *Rag1/2* line is not the result of a more rapid induction of the defenses triggered by individual *Rag* genes.

### Biological processes modified by aphids in each soybean genotype

Based on the unique global response of the *Rag1/2* genotype and its enhanced resistance compared with each of the monogenic genotypes, we hypothesized two scenarios that could be occurring in *Rag1/2* plants. First, it is possible that the synergistic gene set is simply a larger number of genes that function within the same or similar biological pathways as those regulated in either of the monogenic responses. In this case, we would expect to find a large overlap in the type of response (biological processes) in *Rag1/2* when compared to each monogenic genotype. Alternatively, each response, and particularly the synergistic response observed for *Rag1/2*, could regulate unique biological processes that would result in distinct metabolic outcomes.

**Fig. 4:**
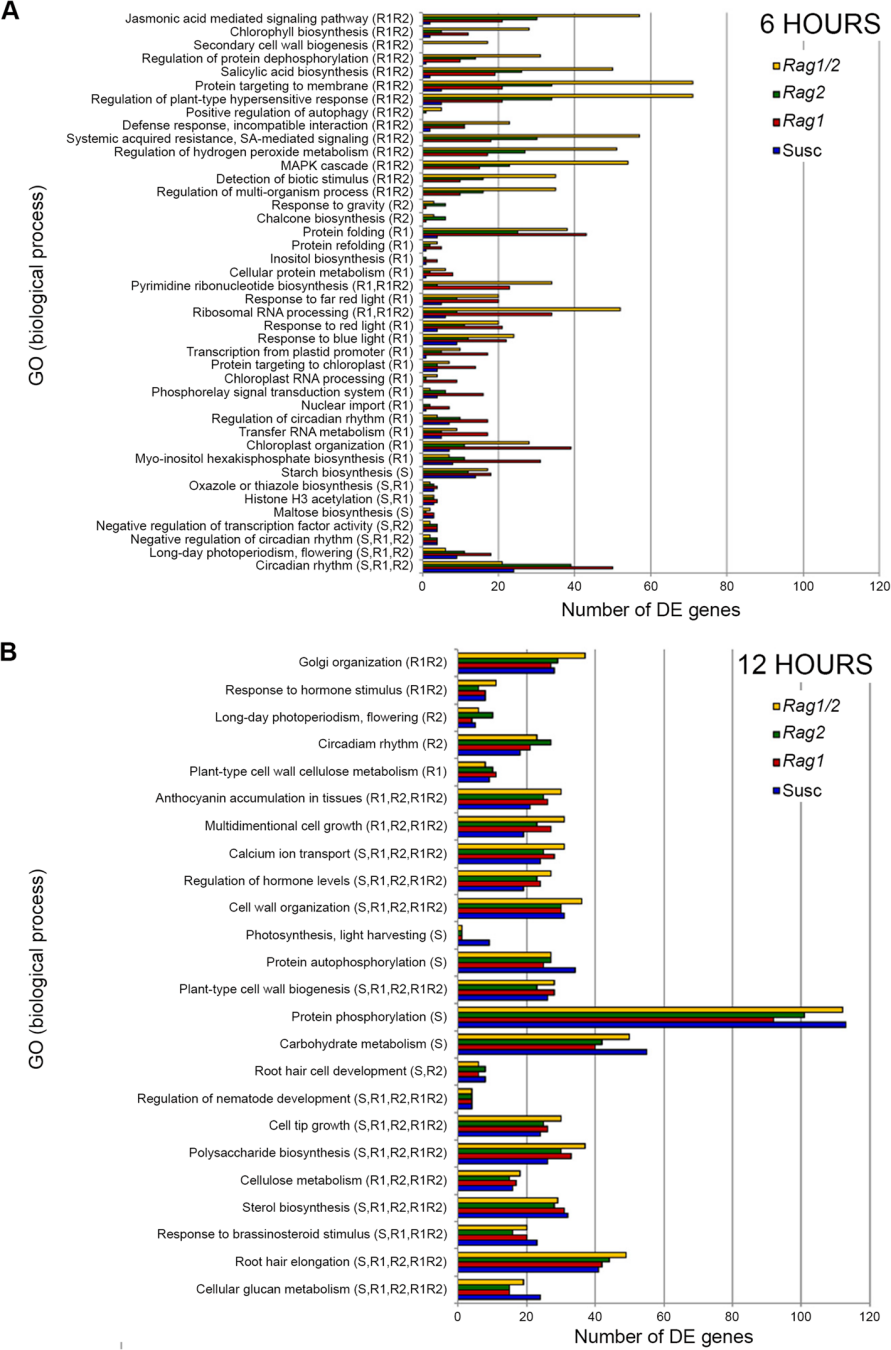
Distinct biological processes are regulated early in the pyramid response to aphids. Comparison of GO terms for biological processes significantly modified after 6 h (**A**) or 12 h (**B**) of infestation by soybean aphids in the susceptible (blue bars), *Rag1* (red bars), *Rag2* (green bars) and *Rag1/2* (yellow bars) soybean genotypes. On the Y axis, soybean genotypes for which specific GO terms (biological process) were significantly overrepresented (*p* ≤ 0.05) are indicated in parenthesis. S: Susceptible, R1: *Rag1*, R2: *Rag2,* and R1R2: *Rag1/2*

To determine the similarities and differences in the biological processes occurring within each resistance response, an analysis of gene ontology (biological processes) terms that were significantly overrepresented at corrected *P* < 0.05 was conducted for each genotype. Many biological processes were significantly overrepresented in only one genotype, and few were shared at 6 h of aphid infestation (Fig. 4A; Additional File 4). The *Rag1/2* response showed a number of overrepresented biological processes related to plant immunity (including phytohormone (JA and SA) mediated signaling, SA biosynthesis, secondary cell wall biogenesis, regulation of plant-type hypersensitive response, regulation of hydrogen peroxide metabolism, defense response - incompatible interaction, systemic acquired resistance, detection of biotic stimulus, MAPK cascade and regulation of multi-organism process, and others). The *Rag1* response uniquely involved biological processes related to chloroplasts (i.e., chloroplast organization, chloroplast RNA processing and protein targeting to chloroplast, and transcription from plastid promoter) and several primary metabolism processes. Chalcone biosynthesis and response to gravity were both uniquely overrepresented in the *Rag2* response. For each of the three aphid-resistant soybean genotypes, the majority of the defense-related transcripts were induced (Additional File 4). In the susceptible response, no defense-related GO term was significantly overrepresented at 6 h. However, the unique susceptible response involved starch and maltose biosynthesis with the DE genes being mostly induced.

Contrary to the findings for the 6 h response, biological processes modified by aphids after 12 h were mostly similar among the four soybean genotypes (Fig. 4B). Eleven out of 24 overrepresented GO terms at the 12 h time point were common to all four soybean genotypes. Of these, five were likely related to plant defense responses, including calcium ion transport, regulation of hormone levels, response to hormone stimulus, cell wall organization and plant-type cell wall biogenesis (Fig. 4B). For most of the common GO terms, the DE genes were repressed in all soybean genotypes (Additional File 4).

Overall, our results showed that the transcriptional responses of the four soybean genotypes at 6 h were mostly unique to each genotype and affected distinct biological processes. Specifically, GO analysis indicated that in the 6 h *Rag1/2* response, the biological functions that were differentially regulated by aphid presence were not significantly overrepresented in the response of the individual *Rag1* or *Rag2* genotypes. On the other hand, at 12 h the transcriptional responses and the biological processes were more similar among the four soybean genotypes.

### Biological processes modified by soybean aphids in the *Rag1/2* synergistic response

To examine the biological processes regulated by the synergistic genes in the *Rag1/2* soybean genotype, GO analysis was conducted for DE genes that were unique to this genotype at each time point. There were 1000 transcripts unique to *Rag1/2*, and 13 biological process terms were significantly overrepresented (Table 2) 6 h after aphid infestation. No significantly overrepresented GO terms were identified in the 12 h response. A number of significantly overrepresented GO terms for the 6 h synergistic response were related to plant defense (secondary cell wall biogenesis, regulation of multi-organism process, detection of biotic stimulus, systemic acquired resistance, SA mediated signaling, and MAPK cascade), and most of the DE genes in these categories were induced by aphids (Table 2). Another group of overrepresented terms were involved in photosynthesis-related biological processes (photosynthesis-light harvesting, light reaction, photosystem II assembly, and chlorophyll biosynthesis), but the majority of these transcripts were repressed in the *Rag1/2* response (Table 2). The majority of the synergistic transcripts included in the defense-related GO categories were distributed in clusters 1, 2 and a few were in cluster 3 in Fig. 3B, and in cluster 2 in Fig. 3C. Most of the photosynthesis-related transcripts, strongly repressed only in *Rag1/2*, were located in cluster 2 in Fig. 3B, and in cluster 1 in Fig. 3C. This further confirmed the uniqueness of the *Rag1/2* response to aphids when compared to the other three soybean genotypes.

**Table 2:** Biological processes differentially represented in the *Rag1/2* synergistic response to soybean aphids after 6 h

GO_id	GO_description (biological process)	Total number of DE genes	Genes induced	Genes repressed
GO:0006364	rRNA processing	41	15	26
GO:0009834	Secondary cell wall biogenesis	17	16	1
GO:0043900	Regulation of multi-organism process	24	16	8
GO:0015995	Chlorophyll biosynthesis	25	0	25
GO:0009595	Detection of biotic stimulus	24	16	8
GO:0000165	MAPK cascade	37	25	12
GO:0009765	Photosynthesis, light harvesting	9	0	9
GO:0006606	Protein import into nucleus	21	21	0
GO:0019684	Photosynthesis, light reaction	23	1	22
GO:0010508	Positive regulation of autophagy	4	4	0
GO:0010207	Photosystem II assembly	25	0	25
GO:0009862	Systemic acquired resistance, SA mediated signaling	38	23	15
GO:0035304	Regulation of protein dephosphorylation	23	4	19

### Hormone signaling in the plant response to soybean aphids

Given the strong representation of phytohormone signaling in our gene ontology results and that the crosstalk among hormone signals is an important component of the *R* gene-mediated response, we identified phytohormone signatures using the Hormonometer tool [45] that compares a given dataset to several Arabidopsis datasets of hormone-regulated gene expression. Since the tool has been developed for Arabidopsis, it was adapted by first identifying the closest Arabidopsis homolog for each DE gene, as has been previously described [46, 47]. Hormonometer results (Additional File 3, Fig. S3), which determine the correlation of a given dataset to a hormone response dataset, indicated that genes associated with the two main defense hormones, JA (MJ, Methyl Jasmonate, for the Arabidopsis datasets) and SA, are upregulated in response to aphids in all four genotypes. The JA response seems to be higher in the resistant genotypes, more specifically in *Rag1/2* and *Rag2* at 6 h, and it tapers down at 12 h. This result agrees with a recent experiment showing accumulation of JA-Ile in *Rag1/2* plants after 6 h of aphid infestation [48]. The SA response seems more sustained through the two time points in all genotypes, although weaker overall in the susceptible line. In contrast, another hormone that is frequently related to defense responses, ethylene (ET), is generally suppressed throughout the period analyzed in all genotypes, with a stronger suppression in the susceptible line. Auxin signaling was observed at 6 h but was less prevalent at 12 h. ABA response was observed at 12 h for all genotypes. Both gibberellin and brassinosteroid signaling were generally repressed, while cytokinin signaling was not prevalent in any genotype. Although it provided an important insight into phytohormone activity during the response to aphids, this analysis failed to identify a phytohormone signaling pattern that could explain the large difference in gene expression observed for the *Rag1/2* line at 6 h after aphid infestation.

### Identification of transcription factors that participate in the *Rag1/2* synergistic response to aphids

**Fig. 5:**
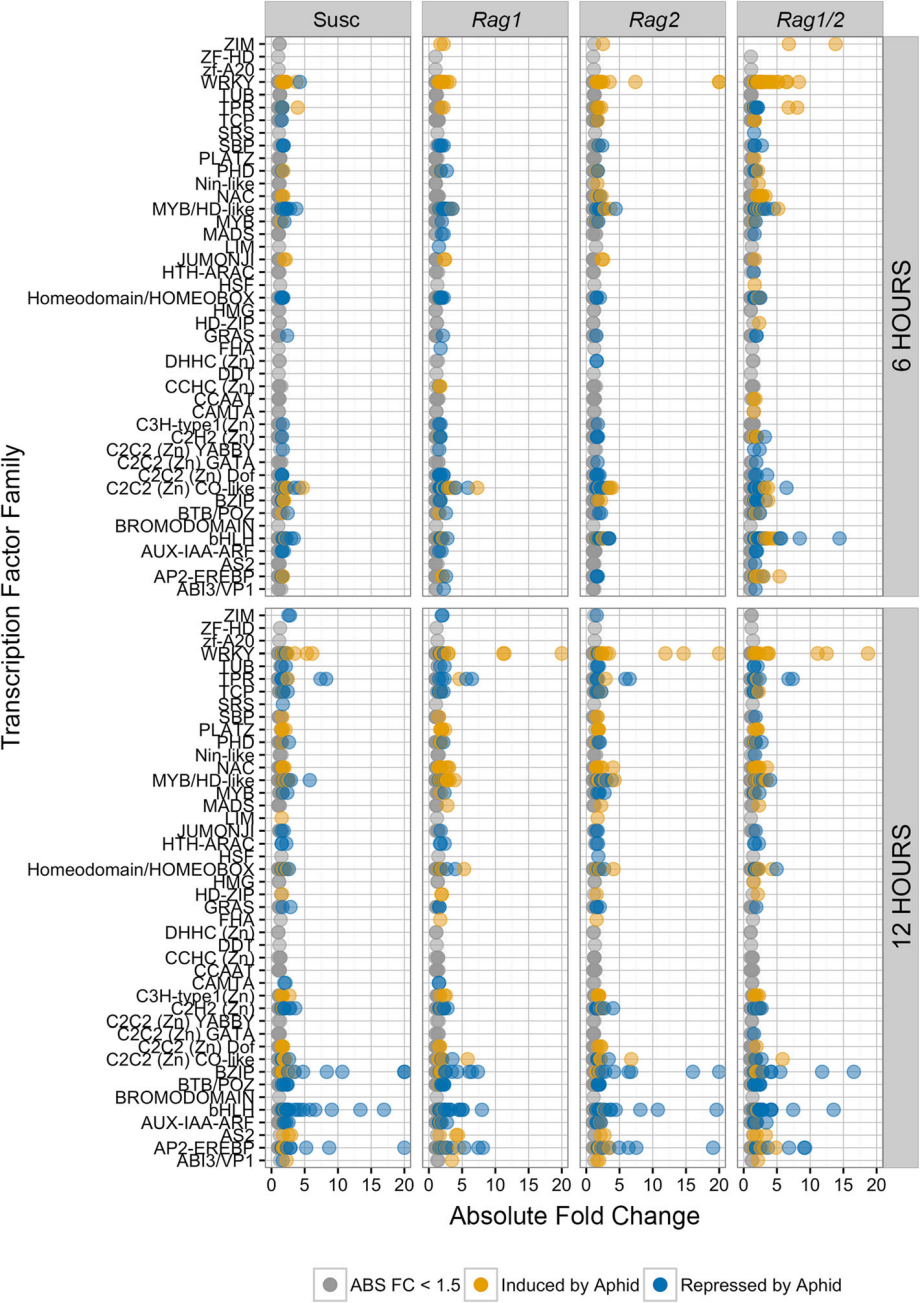
The pyramid response includes a large number of transcription factors (TF). Expression patterns of TF that were differentially expressed between aphid-treated and mock-treated samples for each soybean genotype at both time points were plotted (with and absolute fold change larger than 1.5). The x axis indicates absolute fold change in gene expression, and the y axis shows TF families. Several differentially expressed transcription factors per family are plotted for each comparison

Another important component of a plastic defense response is the presence of transcriptional networks that add regulatory nodes to signaling pathways. We used the SoyDB transcription factor database [49] to identify DE TF in order to determine their participation in the *Rag1/2* synergistic response to soybean aphids. Given the importance of JAZ transcriptional repressors in control of the JA pathway [50–52], JAZ homologs were also added to our analysis even though these proteins do not bind DNA directly. Like the full transcriptome set, TF expression patterns (Fig. 5) exhibited distinct responses among genotypes at 6 h but similar responses at 12 h. Across all treatments, there were 522 TF that responded to infestation by soybean aphids, belonging to 44 TF families.

We identified DE TFs belonging to 33 different families in the 6 h response, with 107, 89, 149 and 43 differentially expressed TF in *Rag1*, *Rag2*, *Rag1*/2 and susceptible genotypes, respectively. We identified individual DE TFs that were strongly differentially expressed in response to aphids and TF families where multiple members respond to aphid feeding (Fig. 5). Ten TF families of interest include: ZIM, WRKY, TPR, NAC, MYB/HD-like, JUMONJI, Homeodomain/HOMEOBOX, C2C2 (Zn) CO-like, bHLH and AP2-EREBP. For most of these families, the magnitude of gene expression is greatest in *Rag1*/2. While many of the TF families had mixed expression patterns, genes in the WRKY and ZIM families were highly induced in the three resistant genotypes. TFs belonging to 39 families were present in the 12 h response. We identified 192, 169, 201 and 152 differentially expressed TF in *Rag1*, *Rag2*, *Rag1*/2 and susceptible genotypes, respectively. Ten TF families responded to aphids at 12 h: WRKY, TPR, NAC, MYB/HD-like, C2H2 (Zn), Homeodomain/HOMEOBOX, C2C2 (Zn) CO-like, BZIP, bHLH and AP2-EREBP (Fig. 5). Unlike the 6 h timepoint, by 12 h after aphid treatment, most of these families respond similarly across all four genotypes.

The difference in *Rag1*, *Rag2* and *Rag1/2* TF expression patterns at 6 h again suggests a synergistic response. To evaluate this further, we identified all TF families significantly over represented within each genotype and time point and within the 6 h and 12 h synergy genes. C2C2(Zn)CO-like TFs were significantly over represented at 6 h in *Rag1* (Corrected *P* < 3.63E-12), WRKY TFs were significantly overrepresented in *Rag2* at 6 h (Corrected *P* < 4.52E-2), and both C2C2(Zn)CO-like (Corrected *P* < 2.72E-4) and WRKY (Corrected *P* < 5.72E-7) TFs were significantly overrepresented in *Rag1*/2 at 6H. The *Rag1/2* genotype had the highest number of DE WRKY TF (23 WRKYs) when compared with *Rag2* (11 DE WRKYs) or *Rag1* (4 DE WRKYs), while in the susceptible control only 1 DE WRKY transcription factor was observed, and this WRKY was repressed (Additional File 5). Remarkably, 13 WRKY TFs (57% of Rag1/2 TFs) were differentially expressed only in *Rag1/2* and are included in the 1000 synergy genes identified at 6 h. WRKY TFs were significantly overrepresented among the 91 TFs found within the 1000 synergy genes (Corrected *P* < 2.53E-3). In contrast, when we examined the C2C2(Zn)CO-like TFs, only two of the 11 (18%) differentially expressed genes in *Rag1/2* appeared synergistic. At 12 h, BZIP TFs were significantly over represented in *Rag1* (Corrected *P* < 1.53E-2), while TCP TFs were significantly overrepresented in *Rag1*, *Rag2* and *Rag1*/2 (Corrected *P* < 3.30E-4, 6.97E-3, and 4.06E-3, respectively). Of the 10 TCP TFs expressed in *Rag1/2* at 12 h, three corresponded to 12 h synergistic genes.

With these examples in mind, we then reexamined the data to identify all TF families at 6 and 12 h where 50% or more of the TFs expressed in *Rag1/2* also corresponded to synergistic genes. This approach identified 19 TF families at 6H (Additional File 6), including WRYKY (57%), BHLH (75%), AP2-EREBP (71%) and NAC (86%) TFs, the major plant TF families involved in regulation of plant defense [11]. Similarly, we identified ten TF families at 12 h with both NAC and WRKY TF familied identified at both 6 and 12 h. Remarkably, this approach also identified ZIM transcription factors as important. While the soybean genome contains 24 ZIM TFs, two (orthologs of AtJAZ1 and AtJAZ2) were unique to *Rag1/2* at 6 h (Fig. 5). JASMONATE-ZIM-DOMAIN (JAZ) TF act as repressors of the JA pathway [53]. Overall, the observed expression patterns for the WRKY and JAZ TF suggests that the *Rag1/2* response is distinct from that of the monogenic genotypes and that for a subset of genes the magnitude of change in expression is larger in the pyramid than in either of the single *Rag* genotypes.

**Fig. 6:**
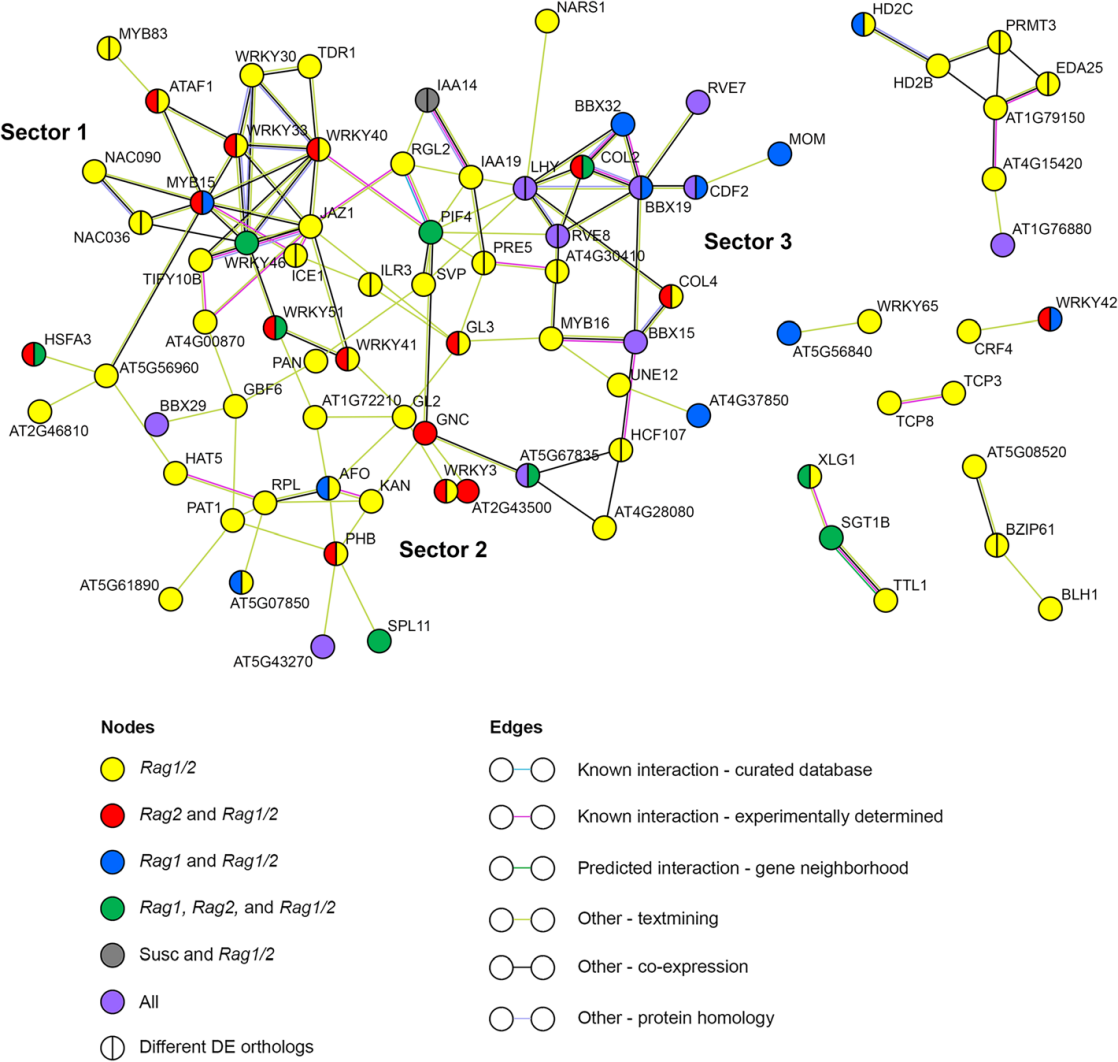
Transcription factor networks in the 6 h pyramid response are regulated by *Rag1* and *Rag2*. Protein-protein interaction network for TF differentially regulated in the *Rag1/2* genotype after 6 h of aphid infestation. The network was built using the closest Arabidopsis homolog for each soybean TF. Colors indicate whether each TF was DE in the pyramid only or in the pyramid and other genotypes in the 6 h response. Split circles indicate more than one soybean homolog corresponding to the same Arabidopsis TF

The large number of TF differentially regulated in the *Rag1/2* response associates well with the observed transcriptional changes in this genotype in response to aphids. However, on its own it does not explain why the pyramid response is so different from the response observed in the *Rag1* and *Rag2* monogenic genotypes. One possibility to explain this observation is that when *Rag1* and *Rag2* are combined, the suite of TF regulated by *Rag1* signaling interact and cooperate with the repertoire of TFs in the *Rag2*-mediated response and combine in unique ways to induce changes in the expression of the synergistic genes. Thus, we could expect to see TF that are shared between *Rag1/2* and each of the individual *Rag* genotypes to act as nodes in a TF network to regulate the synergistic response. To test this idea, we performed a STRING analysis to identify potential interactions among TF differentially expressed in the *Rag1/2* response. Since there is scarcity of soybean-specific experiments incorporated in available databases, we performed this analysis using the Arabidopsis homolog more closely related to each DE soybean TF. After removing TF that did not participate in any interaction, each remaining TF was cataloged to indicate whether it was DE only in the *Rag1/2* response or if it was also DE in other genotypes. Given the ancient hexaploidy nature of the soybean genome [42], several soybean genes can have close homology to a unique Arabidopsis gene. For this annotated network (Fig. 6), if more than one soybean TF corresponding to the same Arabidopsis homolog was present in the *Rag1/2* DE dataset, it is indicated as a split circle. Differences in regulation are indicated with different colors. The analysis identified a large *Rag1/2*-specific TF network with high connectivity and several smaller networks with few genes. Remarkably, of the 82 TFs depicted in Figs. 6, 52 correspond to synergistic TFs.

The large network showed sectors with different levels of influence from individual *Rag* genes (Fig. 6). The expected cooperative contribution from *Rag1* and *Rag2* signaling, represented with blue (genes DE in *Rag1* and *Rag1/2*) and red (genes DE in *Rag2* and *Rag1/2*), is apparent in sector 1, centered around *MYB15*. Two soybean TFs homologous to the *AtMYB15* gene were differentially upregulated, one in *Rag1* and *Rag1/2* (*Glyma.02G005600*, FC = 2 fold and FC = 2.8 respectively) and the other in *Rag2* and *Rag1/2* (*Glyma.20G209700*, FC = 3.6 fold and FC = 5.2 respectively). These TF are connected to other TF DE in *Rag2* and the pyramid (homologs of *AtATAF1*, *AtWRKY33* and *AtWRKY40*) or DE in all resistant genotypes (*AtWRKY46*), and this cluster of TF is then connected to a series of synergistic (yellow) TF. Sector 1 contains TF mainly associated with phytohormone signaling, defense and response to stress, and genes in this sector are mainly induced in the response to aphids. Sector 2 has less connected nodes, but also shows TF that are regulated by *Rag1* or *Rag2* signaling interacting with each other. Particularly interesting are the homologs of *AFO* (two homologs, *Glyma.05G056000* DE in *Rag1* and *Rag1/2*, and *Glyma.17G138200* only in *Rag1/2*) and *PHB* (two homologs, *Glyma.15G129700* DE in *Rag2* and *Rag1/2*, and *Glyma.09G023600* only in *Rag1/2*) that interact with each other and several synergistic TF. The TF in this sector are mainly associated with development of axial symmetry, epidermal cell patterning, and chlorophyll biosynthesis, and expression patterns suggest that these processes are repressed in the pyramid genotype. Sector 3 contains TF involved in circadian regulation and flowering, with many of the genes showing DE in response to aphids in all genotypes, including susceptible plants.

## Discussion

Gene pyramiding has become an important plant breeding and resistance management tool to develop more durable resistant crop cultivars against insect pests and pathogens. Thus, the effect of gene pyramiding has been widely investigated at the phenotypic level. For instance, resistant cultivars containing multiple *R* genes have been developed to effectively protect plants against soybean aphids [17, 36–38], Soybean mosaic virus [54], bacterial blight and blast in rice [55–57], late blight of potato [58, 59], wheat leaf rust [60], and wheat powdery mildew [61]. In many cases, the combination of *R* genes in an individual genotype results in enhanced resistance when compared to genotypes with one *R* gene (for a summary see [14]). However, few reports have studied the molecular bases for the increase in resistance observed in *R* pyramids.

Recently, Kamphuis et al. [16] analyzed the effects of pyramiding *AKR* and *AIN*, two *R* genes that confer resistance to the bluegreen aphid (BGA) in *Medicago*. Pyramiding these genes in a single genotype results in enhanced resistance to BGA, while at the same time the pyramid eliminates deleterious effects associated with *AIN*. The analysis identified an important crosstalk among JA and SA signaling and regulation of reactive oxygen species (ROS) and cell death associated with *R* responses. In response to BGA attack, plants carrying only *AIN* deploy a strong hypersensitive response with cell death mediated by SA and ROS. On the other hand, the response in plants carrying *AKR* shows a unique JA-mediated response (not observed in basal defense or in the *AIN* genotypes). The pyramid genotype responded to aphids with a significant increase in JA and SA signaling that seem to act cooperatively to induce a stronger defense response against BGA. However, it is possible that JA also acts to reduce the hypersensitive response driven by *AIN,* thus eliminating negative effects associated with widespread cell death [16]. In a different study, Gao et al. [62] analyzed the effect of pyramiding *Xa21* and *xa5*, two *R* genes that provide resistance to bacterial blight in rice. This study used a genome-wide approach to identify transcriptional changes associated with the presence of individual *R* genes or a pyramid in a single genotype; however, the analysis was conducted in the absence of the pathogen. Phenotypic analysis indicated that the pyramid had higher level of resistance to bacterial blight than either of the single *R* gene lines. Transcriptome data identified 2367, 2412, and 3596 DE genes in the NILs carrying *Xa21*, *xa5*, and *Xa21* + *xa5* respectively, when compared against the susceptible control line. Since no pathogen was present in these experiments, it is clear that in the case of the rice *R* genes there is a constitutive defense component associated with resistance. In addition, although there was a large number of genes (1633) DE only in the pyramid line, the focus of the study centered on the DE genes common to all *R* lines [62]. Thus, a deeper mechanistic understanding of the molecular bases for enhanced resistance in the pyramid was missed.

Pyramiding the *Rag1* and *Rag2* genes confers enhanced resistance to soybean aphids. Previous work indicated that, in plants carrying either of the two genes individually, a differential transcriptional response occurs early in the interaction with the insect [22–24]. We observed that the transcriptome response of the monogenic *Rag1* and *Rag2* genotypes were dissimilar to each other at 6 h after aphid infestation. Moreover, the *Rag1/2* pyramid had its own strong, distinct response to aphids at this early time point. Interestingly, at 12 h, the *R* gene-mediated response of the monogenic genotypes and the pyramid were more similar. This suggests that events occurring during the first few hours after the initiation of aphid infestation are responsible for the enhanced resistance seen in the pyramid line. While some additive effects were encountered, the differential response observed in the pyramid is not purely the sum of the transcriptional responses observed in each monogenic genotype, indicating a synergistic interaction between *Rag1* and *Rag2* signaling. The cluster dendrogram showed that the *Rag1/2* transcriptional response to aphids was more similar to that of the *Rag2* genotype at 6 h but closer to the *Rag1* response at 12 h, suggesting that modification of transcripts associated with the *Rag2* resistance likely occurs earlier compared to transcripts involved in the *Rag1* response in the pyramid genotype. This result suggests some hierarchical order in the *Rag1/2* response, with *Rag2* having more influence on the early response, although the majority of the DE genes observed at 6 h in the pyramid genotype are exclusively regulated in this genotype.

The *Rag1/2* pyramid does not seem to follow the patterns observed in the *R* pyramid studies described above. We did not observe unique phytohormone signatures in the pyramid, and transcriptome differences between the pyramid and monogenic genotypes in the absence of aphids were minor. However, we observed a large transcriptional reprograming unique to the pyramid genotype. This synergistic response, defined as a set of genes DE only in the pyramid and not in either the Rag1 or Rag2 genotypes, is rapidly mounted, mostly evident at 6 h after the beginning of aphid infestation, and it affects a series of unique biological processes that may increase the defensive output in this genotype resulting in the enhanced resistance phenotype observed. As an example, our TF analyses suggest that, upon activation, *Rag1* and *Rag2* induce expression of different TF that cooperate on specific promoters to trigger this unique response. Many of the targets are also TF, creating an amplification of this synergistic effect.

Additionally, many TF act as homo or heterodimer complexes. Aphid-dependent induction of several highly similar soybean TF, close homologs to the same Arabidopsis TF, would provide greater complex diversity, creating novel target specificities. A similar scenario has been described for Arabidopsis allotetraploids created by crossing *A. thaliana* and *A. arenosa* autotetraploids [63]. In the allotetraploid progeny, differential accumulation of specific homoeologous TF (*AtWRKY18*, *AaWRKY40*, and *AtWRKY60*) was observed in response to bacteria and SA treatments. Changes in protein-protein interactions resulted in the formation of different heterodimers and these different complexes showed altered affinity for target promoters. These changes cause altered regulatory networks that could explain the enhanced resistance observed in the allotetraploids [63]. In the TF regulatory network identified in the *Rag1/2* pyramid, two hubs with concerted action of “parental” TF, that is, TF DE in one of the monogenic genotypes and the pyramid, were predicted. One sector is centered on the interactions among homologs of *MYB15*, *WRKY33*, *WRKY40*, and *WRKY46* (Sector 1, Fig. 6); the other is centered on the *AFO-PHB* hub (Sector 2, Fig. 6). In both cases, each node represents two or more soybean homologs of a unique Arabidopsis gene, suggesting the potential for a diversity of TF complexes. These hubs may in turn control the phenotypic outputs that increase the pyramid resistance against aphids, such as the biological processes uniquely regulated in the early (6 h) pyramid response that include defense responses related to increase in secondary cell wall deposition and suppression of photosynthesis.

While the TF network presented here is based on homology to Arabidopsis genes, and thus has limited power due to the uncertainty of assigning orthologs and potential functional divergency, it allows as to make predictions that can direct future functional studies.

The MYB-WRKY hub is likely to participate in the establishment of the early defense responses of the pyramid genotype. Cooperation of MYB and WRKY TF on individual promoters has been shown for the activation of defense against herbivores in soybean [64], and the roles of Arabidopsis MYB15, WRKY40, WRKY46, and WRKY33 in defense responses, including defense against aphids, are well-documented [65–70]. In fact, these four TF were among a set of TF specifically induced by cabbage aphid feeding in an experiment comparing Arabidopsis responses to insect and bacterial attacks [71], AtMYB15 is necessary for full harpin-induced resistance to green peach aphid in this plant [72], and silencing a wheat homolog of *AtWRKY33* results in reduced resistance to the Russian wheat aphid [73]. Five soybean homologs of *AtWRKY40* are induced in the early pyramid response to aphids in our dataset. Two of those (*Glyma.17G222300* and *Glyma.14G103100*) are also induced in the *Rag2* response, while the other three (*Glyma.06G061900, Glyma.15G003300*, and *Glyma.07G023300*) are only expressed in the synergistic set. We had previously observed increase of *Glyma.17G222300* expression as part of the soybean PTI response to long-term aphid feeding [41]. In addition, *Glyma.17G222300* and *Glyma.14G103100* are also among the most highly induced genes in the resistance response to soybean cyst nematode (SCN) in wild soybean [74], and overexpression of *Glyma.14G103100* resulted in decreased susceptibility to soybean cyst nematode in the susceptible soybean cultivar Williams 82 [75]. Two homologs of *AtMYB15* are DE in the pyramid 6 h set, *Glyma.20G209700* (also induced in *Rag2*) and *Glyma.02G005600* (also induced in *Rag1*). *Glyma.20G209700* is also upregulated in the PTI response to long term aphid feeding [41]. *Glyma.02G005600* is also induced in the resistant response to SCN [74], suggesting that the MYB-WRKY hub also participates in the resistance response to other herbivores.

Cell wall modifications are a significant component of plant-insect interactions [76–78], and our analysis indicated that secondary cell wall biosynthesis is differentially regulated in the pyramid 6 h synergistic response. MYB TF are important regulators of the phenylpropanoid pathway and lignin biosynthesis [69]. AtMYB15 acts as a positive regulator of defense-induced lignification in Arabidopsis by binding to promoters of genes involved in G-lignin biosynthesis, and plants overexpressing *AtMYB15* had increased lignin content [79]. Similarly, overexpression of *CmMYB15* resulted in an increase in lignin accumulation in chrysanthemum and increased resistance to the aphid *Macrosiphoniella sanborni* [80]. Another important MYB TF in the regulation of secondary cell wall biosynthesis is AtMYB83 [81, 82], and two homologs of *AtMYB83* (*Glyma.11G133700 and Glyma.12G057900*) are induced in the 6 h synergistic response and are part of the MYB-WRKY hub. Interestingly, homologs of several direct targets of AtMYB15 and AtMYB83 are differentially regulated in the pyramid at 6 or 12 h after aphid infestation, including homologs of *PAL1*, *C4H*, and *COMT* (MYB15 targets [79]), and *MYB43*, *PAL1*, *MYB32*, Family 8 and Family 43 glycosyltransferases, and *EXPL1* (MYB83 targets [82]). Heterologous expression of *Medicago truncatula WRKY108715* (*AtWRKY33* homolog, and closest homolog to the synergistic gene *Glyma.09G280200* in the MYB-WRKY hub) caused a large increase in lignin content in transgenic tobacco plants [83]. Thus, regulatory interactions between MYB and WRKY TF could be a significant contribution to cell wall strengthening which could limit the ability of aphids to penetrate leaf tissues to reach the phloem and in this way increase resistance in the pyramid genotype.

As mentioned, MYB TF are important regulators of the phenylpropanoid pathway which is also responsible for the biosynthesis of isoflavonoids in soybean, and quantitative trait locus analyses showed a correlation between resistance to soybean aphid and isoflavone content [84]. In a previous experiment, we showed that isoflavones can act as deterrents for soybean aphids [41]. Thus, another interesting outcome of MYB regulation is the production of isoflavones. One of the *MYB15* homologs in MYB-WRKY hub, *Glyma.20 g209700* (induced in *Rag2* and *Rag1/2*) was identified in a genome-wide association study as one of the loci significantly associated with isoflavone concentration (called *GmMYB29* in that study) and overexpression and RNAi of these gene resulted in increased and decreased isoflavone content respectively when compared to controls, in experiments using transgenic soybean hairy roots [85]. The other *MYB15* homolog, *Glyma.02G005600* (induced in *Rag1* and *Rag1/2*) has also been implicated in the regulation of isoflavone biosynthesis. This TF, called GmMYB29A2 in the study, specifically controls glyceollin I biosynthesis in response to the oomycete *Phytophthora sojae* [86]. Moreover, several transcripts corresponding to the isoflavonoid branch of the phenylpropanoid pathway are also induced in the pyramid at 6 or 12 h, including isoflavone reductase (*Glyma.11G070600*), isoflavone 2′-hydroxylase (*Glyma.11G051800*), 2-hydroxyisoflavanone dehydratase (*Glyma.10G250300*), and isoflavone 4′-O-methyltransferase (*Glyma.13G173300* and *Glyma.13G173600*). Thus, it appears that both the *Rag1* and *Rag2* pathways contribute to isoflavone production in the pyramid genotype and these phytoalexins could also be part of the defense output contributing to the increased-resistance phenotype observed for this genotype.

Suppression of photosynthesis and chlorophyll biosynthesis are also differentially regulated by aphid infestation in the synergistic response at 6 h. AtWRKY40 acts as a negative regulator of expression of nuclear genes that encode chloroplast proteins [87]. Specific AtWRKY40 targets include genes corresponding to light-harvesting chlorophyll a/b-binding (LHCB) proteins and a glutamyl-tRNA reductase involved in chlorophyll synthesis (HEMA1) [87, 88]. In addition to the *WRKY40* homologs identified in the pyramid 6 h response, several photosynthesis-related WRKY40 targets are significantly repressed in the synergistic response, including homologs of *LHCB1.4* (*Glyma.16G165200*, *Glyma.16G165500*, *Glyma.16G165800*, *Glyma.16G162600*), *LHCB3* (*Glyma.02G080800*), *LHCB6* (*Glyma.05G119000*), and *HEMA1* (*Glyma.06G091600*, *Glyma.14G185700*, *Glyma.02G218300*). Thus, the MYB-WRKY hub identified in the 6 h pyramid response could also regulate the repression of photosynthesis observed in the early pyramid response. However, hubs in other sectors of the TF network identified in the 6 h pyramid response may also participate in the negative regulation of photosynthesis. A homolog of *GNC* (*GATA, nitrate-inducible, carbon metabolism-involved*) is repressed in the pyramid [*Glyma.13G103900* (repressed in *Rag2* and *Rag1/2*), Sector 2 in Fig. 6]. GNC controls expression of chloroplast genes and chlorophyll production in Arabidopsis, and *gnc* mutants have reduced levels of chlorophyll [89, 90]. Specific targets of GNC [91], such as *LHCB1.4*, are among the genes repressed in the 6 h synergistic set. Phytochrome A signal transduction (*PAT1*), a positive regulator of *LHCB* and *HEMA1* expression [92] is also part of the sector 2 network (*Glyma.12G216100,* synergistic gene) and is repressed at 6 h. In addition, TF in sector 3 that include light-regulated and circadian clock-responsive factors could also participate in the photosynthesis regulation observed in the pyramid response [93].

Repression of photosynthesis is commonly associated with ETI responses [94–97]. Current models indicate that these changes in photosynthesis result in increases in reactive oxygen species (ROS) that are produced due to uncoupling of the electron transfer chain. The oxidative burst associated with these ROS is a key component of the ETI response, contributing to the hypersensitive response and other signaling functions, cell wall strengthening, and direct effects on pathogens [98]. Activation of the MPK3/MPK6 signaling cascade is critical for photosynthesis inhibition, ROS production, and ETI [97]. While the role of chloroplasts has not been well-characterized, ROS participation in defense responses in plant-aphid interactions have been previously reported [16, 22, 99–102]. In our analysis, a strong suppression of photosynthesis is observed in the early pyramid synergistic response. In addition, “regulation of plant-type hypersensitive response”, “regulation of hydrogen peroxide metabolism”, and “MAPK cascade” are among the biological processes overrepresented only in the *Rag1/2* genotype dataset at 6 h (Fig. 4), and *MPK3* (*Glyma.U021800*) is induced only in the *Rag1/2* line at this time point. Thus, the suppression of photosynthesis observed in the *Rag1/2* genotype may be associated with the production of ROS that could contribute to a hypersensitive-type response and also to cell wall-strengthening.

The differential *Rag1/2* response appears transient, that is, a large number of genes DE at 6 h in this genotype return to basal levels by 12 h. A similar rapid and transient response to aphids was observed for *Rag5*-containing soybean plants when compared to the response of a susceptible NIL [43]. In that case, a large number of genes is DE in the infested *Rag5* line at 6 or 12 h after aphid infestation when compared with the susceptible line, but only a few genes are DE between lines at 48 h post-infestation, indicating that the resistance response is rapid and may be established between 6 and 12 h after aphid infestation, as previously suggested [22]. Similarly, the susceptible and resistant response of peach plants to green peach aphid infestation show low level of similarity (23%) at 3 h post-infestation but a much larger overlap (59%) at 12 h [103]. It is also important to note that while gene expression may have subsided by 12 h, the proteins encoded by genes DE in the early pyramid response could have long half-lives, and many of the changes triggered by the pyramid response, such as strengthened cell walls or accumulation of phytoalexins, could persist for a longer period of time.

## Conclusions

Pyramiding resistance genes is an effective method for crop improvement, but the molecular mechanisms underlying the enhanced resistance phenotype are not well-understood. We found that individual aphid resistance genes regulate different subsets of the defense repertoire in soybean, and that combining two *R* genes that confer resistance to soybean aphids in the same genotype results in an unexpected synergistic effect. The *R* gene-mediated response of monogenic genotypes and the pyramid are mostly different early (6 h after initial aphid infestation) but become more similar to each other and the susceptible response after 12 h, suggesting that the mechanisms that contribute to resistance are established very rapidly in this system. The unique, large synergistic response observed in the pyramid is likely responsible for the improved resistance phenotype expressed in this genotype, and it seems to be the result of a transcriptional network that combines *Rag1*- and *Rag2*-dependent TF in central interaction hubs that control target genes, including other TF, differentially regulated only in *Rag1/2* plants. Our results suggest that the pyramid genotype elicits a defense response that is the combination of additive and synergistic effects. These results could explain some of the genetic variation observed in different soybean aphid biotypes. In order to overcome the unique response deployed by *Rag1/2*, aphids would need to evolve virulence factors that can overcome not only the individual *Rag1* and *Rag2* mechanisms, but also the *Rag1/2* synergistic response. This is in part corroborated by the recent finding that the genome structure (SNPs) of soybean aphid biotype 4 (virulent on *Rag1*, *Rag2*, and *Rag1/2*) is not simply the additive effect of biotype 2 and biotype 3 (virulent on *Rag1* and *Rag2*, respectively), but rather the majority of the sequence variation in its genome was unique to biotype 4 [104]. This may suggest that alternative mechanisms are needed for overcoming pyramided resistance. The observation of additive and synergistic responses seen in *Rag1/2* soybeans is highly beneficial from a resistance management perspective, as soybean aphids need to overcome three defense mechanisms (*Rag1*, *Rag2*, and the synergistic response), which could greatly extend the durability of these varieties. This synergistic interaction of *R* gene signals causes photosynthesis perturbances that produce ROS and contribute to a rapid hypersensitive-type response, production of feeding-deterrent compounds, and strengthening of secondary cell walls that build up rapid barriers to aphid feeding and establishment of successful colonization. Understanding the specific subsets of defense responses activated by individual *R* genes and the way they interact when combined in the same plant genotype can lead to more effective approaches to biotechnological crop improvement and plant breeding as well as preservation of resistance traits.

## Methods

### Plant and insect material

Four soybean genotypes developed at Iowa State University were used for this study. The soybean genotypes were: IA3027RA12, the *Rag1/2* soybean genotype that has both the *Rag1* and *Rag2* aphid resistance genes; IA3027RA1 contains *Rag1* alone; IA3027RA2 has *Rag2* alone; and IA3027, the susceptible control that has no aphid resistance gene. These soybean genotypes were developed using a backcrossing scheme in which the *Rag1* and *Rag2* donors were A08–123074 and LD08-89051a respectively, with the aphid-susceptible genotype, IA3027 used as the recurrent parent. Soybean genotypes IA3027, IA3027RA1, and IA3027RA12 are approximately 93.75% genetically identical [105, 106]. The genotype with *Rag2* is an experimental line sharing 75% of it genes with the recurrent parent IA3027 [17]. For all experiments conducted in this study, soybean aphid biotype 1 was used, which is unable to colonize soybean genotypes with any known *Rag* gene. Aphids were obtained from a laboratory-maintained colony. Prior to use, aphids were raised on IA3027, the susceptible control. The growth chamber for the aphid colony was maintained at a constant temperature of 25 °C and photoperiod of 16 h light, 8 h darkness.

### Experimental design, treatments and sample collection

Two main sets of experiments were conducted for this study: a) Phenotyping experiments to confirm expected aphid populations for each soybean genotype, and b) Transcriptome response to aphid feeding. All experiments were conducted in growth chambers maintained at a constant temperature of 25 °C and photoperiod of 16 h light, 8 h darkness. To eliminate seed or soil-borne pathogens, seed were surface sterilized with chlorine gas for 16 h as described by Paz et al. [107], and soil used for all experiments was steam-sterilized Metro Mix 900 growing mix (Sun Gro Horticulture, Vancouver, BC, Canada).

#### Phenotyping experiments

To confirm the aphid resistance/susceptibility of each soybean genotype, two no-choice growth chamber experiments arranged in a completely randomized design, with three replicates (pots) for each genotype were conducted. Seeds were individually grown in 5.5″ by 6″ plastic pots. Plants were fertilized once a week with a 1:1 mixture of all-purpose Scott’s Miracle-Gro Excel (21–5-20, The Scott’s Company LLC, Marysville, Ohio, USA) and Cal-Mag Miracle-Gro Professional (15–5-15, The Scott’s Co.), applied at a rate of 12.5 mL/L water. When plants reached the V3 growth stage [108], 30 mixed-age apterous aphids were placed on the abaxial side of the V3 middle leaflet of each plant using a fine paint brush and clip cages were placed on the infested leaflet to restrict aphid movement. One week later, aphid populations on each plant were determined. The phenotypic response of the different soybean genotypes was compared with Student’s *t*-test.

#### RNA-seq experiment and tissue collection

After confirmation of the expected aphid phenotypes, soybean plants of all four genotypes were grown in five growth chambers using a completely randomized design. All chambers were set to similar light, temperature and relative humidity conditions (Light: ~ 350 umol/m2/s, Temperature: 25 °C, Relative humidity: ~ 50%), and planting was done as described previously. Mock plants were grown in separate growth chambers from aphid-treated plants to avoid the potential effects of priming [23]. All plants were staked using bamboo stakes to keep them upright. When plants reached the V3 growth stage, clip cages were placed on the V3 middle leaflet of mock plants in each growth chamber, but without aphids. Thirty mixed-age apterous soybean aphids were placed on the abaxial side of the V3 middle leaflet of aphid-treated plants in each growth chamber and clip cages placed on the leaflet to restrict aphid movement. V3 middle leaflet samples (mock or aphid-treated) were individually collected from each plant after 6 h, gently removing aphids from each leaflet, and flash-frozen in liquid nitrogen. The collected leaflets of mock plants were gently brushed as well to mimic removal of aphids. Twelve hours after introduction of aphids to plants, leaf samples were collected from a separate set of plants. Three plants were used for each sample (genotype x treatment x time), and three replicates were used for the experiment. In total, 144 samples were collected for this study. Prior to processing, all samples were stored at − 80 °C.

### RNA sample preparation and RNA-seq

All flash frozen leaf samples were ground using a mortar and pestle in liquid nitrogen. RNA was extracted from individual samples using the Qiagen® RNeasy® Plant Mini Kit (Qiagen®, Germantown, MD) with some modifications to the manufacturer’s protocol. Samples were incubated at 56 °C for two minutes and vortexed occasionally to disrupt tissue. Additionally, three rounds of RPE buffer washes were done instead of two. RNA samples were DNase treated using the Ambion® TURBO DNA-free kitTM (Ambion®, Austin, TX) to remove genomic DNA contamination. DNase-treated RNA samples were cleaned with Qiagen® RNeasy® MiniElute Cleanup Kit (Qiagen®, Germantown, MD). To ensure the integrity of RNA samples, all samples were checked using an Agilent® 2100 BioanalyzerTM (Agilent®, Santa Clara, CA) and samples with RNA Integrity Number (RIN) of ≥6.9 considered to be of good quality for RNA-seq. RNA from three leaflets (obtained from three different plants) was pooled in equal amounts for each RNA pool to be analyzed. A total of 48 RNA pools (3 replicates × 2 time points × 2 treatments × 4 soybean genotypes) were submitted to the DNA facility at Iowa State University for multiplex library preparation and single end sequencing using the Illumina HiSeq 2500 instrument. Each of the 48 cDNA libraries was sequenced at a read length of 100 base pairs.

### Bioinformatics and statistical analysis

Each of the 48 sequenced libraries were processed using bioinformatics tools. First, the 100 base pair reads were trimmed to remove adapter sequences using scythe (https://github.com/vsbuffalo/scythe). Next, 15 bases (sequencing artifacts) were removed from each read using FASTX trimmer (http://hannonlab.cshl.edu/fastx_toolkit/). Sickle (https://github.com/najoshi/sickle) was then used to remove bases with low quality scores and short reads. Using default settings, TopHat version 2.0.3 [109] was used to align the processed single end reads to the Williams 82 reference genome obtained from Phytozome.net [42]. Samtools view [110] was used to remove unreliably mapped reads (mapping score < 1). The resulting mapping files (bamfiles) were then imported into the R statistical package (R Development Core Team, 2014; http://www.R-project.org/) using the RSamtools statistical package in R (http://bioconductor.org/packages/release/bioc/html/Rsamtools.html). The 48 bamfiles are available from the National Center for Biotechnology Sequence Read Archive under the BioProject accession number PRJNA478017.

The gene feature file for version 2 of the soybean genome was imported to R using rtracklayer [111] and the number of reads aligning to each gene for each sample was determined using GenomicAlignments [112]. Genes with counts per million < 1 were eliminated from further analysis. Data normalization across all treatments and genotypes was done for each time point using the Trimmed Mean of M (TMM) values [113] in the Bioconductor package edgeR [114]. Specifically, edgeR was used for single factor, pairwise comparisons to calculate normalization factors, estimate tagwise dispersion and determine differential gene expression. Initially, our data analysis pipeline used all samples in the experiment. Within the pipeline, we used the graphics package, ggplot2 [115] to visualize normalized gene expression of sample replicates for each treatment to ensure replicability. Due to considerable variation from other replicates, two samples from the *Rag1/2* soybean genotype for the 6 h time point (one mock-treated sample and one aphid-treated sample) were removed from the analysis, leaving two replicates for each of these treatments for this genotype and time point. The design statement in the analysis pipeline was modified to reflect removal of these samples and the process from normalization to identification of DEGs was repeated. To identify genes differentially expressed in response to treatment, genotype or treatment x genotype interaction, our model took into account genotype and treatment (model.matrix(~genotype + trt, Design). To identify genes responding to treatment in a given genotype, our model grouped samples by type (model.matrix(~ 0 + Groups) and then we used contrast statements to facilitate comparisons. In all comparisons, differential expression for each gene was considered significant if false discovery rate (FDR) was < 0.05 (q-values < 0.05). Both treatment and genotype effects were examined in our statistical analysis, identifying genes that responded to aphid treatments in all four soybean genotypes and genotype-specific responses to soybean aphids. Genes with positive fold changes were induced by aphids while genes with negative fold changes were repressed.

Gene list comparisons and construction of Venn diagrams was done using Venny^2.1^ (http://bioinfogp.cnb.csic.es/tools/venny/). Cluster analysis was conducted to identify gene sets with similar expression patterns in response to genotype and treatment. Log counts per million for each sample were used for cluster analysis. DEGs identified at 6 and 12 H were included to examine expression across time, even though a given gene might only be significant at a single timepoint. The hclust package in R (https://www.r-project.org/), with the Pearson correlation and complete linkage methods, was used to construct the heatmap and cluster dendrogram, both of which showed similarities in expression patterns of DE genes across all comparisons. Z-scores for each DE gene were used during clustering.

To compare observed versus expected additive values for 6 h *Rag1/2* synergistic genes, expected values were calculated as the sum of the logFC observed in the *Rag1* and the *Rag2* genotypes. Expected and observed values for the whole synergistic dataset or for induced and repressed subsets were compared using a χ^2^ test.

For comparison of the *Rag1* locus between versions 1 and 2 of the Williams 82 genome assemblies, WebACT [116] was used to run BLASTN [117] alignments of the two regions, requiring 99% sequence identify for homology calls.

### Annotation and GO enrichment of differentially expressed genes

Gene annotation was performed using the gene annotation lookup tool in SoyBase [118] (https://soybase.org/genomeannotation/). The annotation result contained the best UniRef100 hit [119], the best homolog hit in *Arabidopsis thaliana*, and gene ontology (GO) information of the best Arabidopsis homolog (The Arabidopsis information resource, TAIR; https://www.arabidopsis.org/). The GO term enrichment tool in SoyBase (https://soybase.org/goslimgraphic_v2/dashboard.php) was used to identify significantly overrepresented GO terms (biological process) in different gene sets. GO term enrichment was conducted using Fisher’s Exact Test and Bonferroni correction for multiple comparisons, identifying significantly overrepresented biological processes relative to the Williams 82 reference genome in each comparison (adjusted *P* ≤ 0.05).

### Transcription factors (TF), TF expression network, and hormone signal analyses

To identify TF DE in the response to soybean aphids, we used the SoyDB transcription factor database [49]. TFF enrichment was conducted using Fisher’s Exact Test and Bonferroni correction for multiple comparisons, identifying significantly overrepresented TFFs relative to the Williams 82 reference genome in each comparison (Corrected P ≤ 0.05). Transcription factors that were involved in regulation of aphid-responsive biological processes were identified for each comparison for all soybean genotypes tested. Network analysis was performed using STRING with default parameters [120], but using the best Arabidopsis homolog for each soybean gene, identified in the annotation process. Manual curation of the output was carried out to annotate each gene with information of the datasets in which it was found to be differentially expressed, as well as the presence of multiple DE soybean genes corresponding to the same Arabidopsis gene. Changes in hormone signaling were evaluated using Hormonometer [45], also using the best Arabidopsis homolog for each soybean gene, identified in the annotation process, as previously described [46, 47].

## Supplementary Information

**Additional File 1.** Datasets corresponding to all differentially expressed genes in response to aphids for the two time points analyzed (Tab 1: full dataset; tab2: 6 h synergistic response)

**Additional File 2.** List of differentially expressed genes in comparisons among mock-treatments for all genotypes

**Additional File 3. **Supplemental Figs. S1, S2 and S3. Fig. S1. Comparison of expected versus observed log2FC for the *Rag1/2* 6 h synergistic genes. Fig. S2. Comparison of the transcriptional response of each genotype at different time points. Fig. S3. Plant hormone signatures inferred by Hormonometer from the transcriptome responses of each genotype

**Additional File 4.** List of overrepresented GO-terms (biological processes) for each genotype response to aphids for the two time points analyzed

**Additional File 5. **List of *WRKY* transcription factors differentially expressed in response to aphids in all genotypes

**Additional File 6.** Analysis of the enrichment of TF families in the 6 h synergistic response

## Data Availability

All data (raw reads and bamfiles) for this study are available in the National Center for Biotechnology Sequence Read Archive (SRA) under the BioProject accession number PRJNA478017.

## Abbreviations

BGA: bluegreen aphid
DE: differentially expressed
ETI: effector-triggered immunity
FC: fold change
FDR: false discovery rate
GO: gene ontology
JA: jasmonate
NLR: nucleotide-binding leucine-rich-repeat protein
PTI: PAMP-triggered immunity
R: resistance
ROS: reactive oxygen species
SA: salicylate
SCN: soybean cyst nematode
TF: transcription factor
