# Supplementary material for: Interaction between Rag genes results in a unique synergistic transcriptional response that enhances soybean resistance to soybean aphids
Source: BMC Genomics. 2021 Dec 11;22:887. doi: 10.1186/s12864-021-08147-3 (PMC8665634; doi:10.1186/s12864-021-08147-3)
Supplement: Supplementary file 3 — Additional File 3. Supplemental Figs. S1, S2 and S3. Fig. S1. Comparison of expected versus observed log2FC for the Rag1/2 6 h synergistic genes. Fig. S2. Comparison of the transcriptional response of each genotype at different time points. Fig. S3. Plant hormone signatures inferred by Hormonometer from the transcriptome responses of each genotype [file 12864_2021_8147_MOESM3_ESM.pdf]

Supplemental File 3

Supplemental Figures S1, S2 and S3.
Figure S1. Comparison of expected versus observed log2FC values for the *Rag1/2* 6h synergistic genes.
Figure S1. Comparison of the transcriptional response of each genotype at different time points.
Figure S2. Plant hormone signatures inferred by Hormonometer from the transcriptome responses of each genotype.

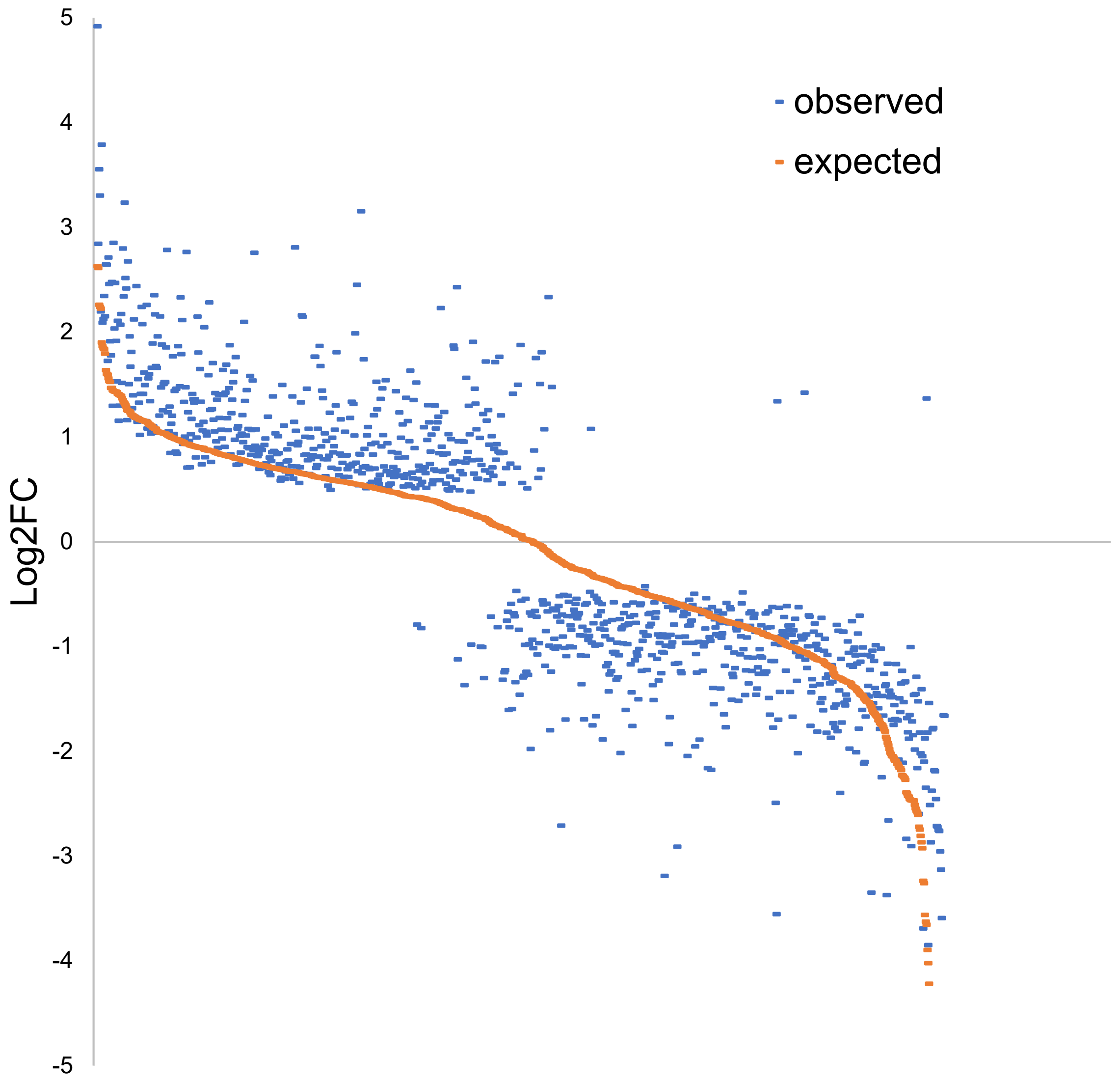

Figure S1. Comparison of expected versus observed log2FC values for the *Rag1/2* 6h synergistic genes. Expected additive values for the *Rag1/2* 6h synergistic gene set was calculated by adding the log2FC corresponding to each gene in the *Rag1* and *Rag2* response.

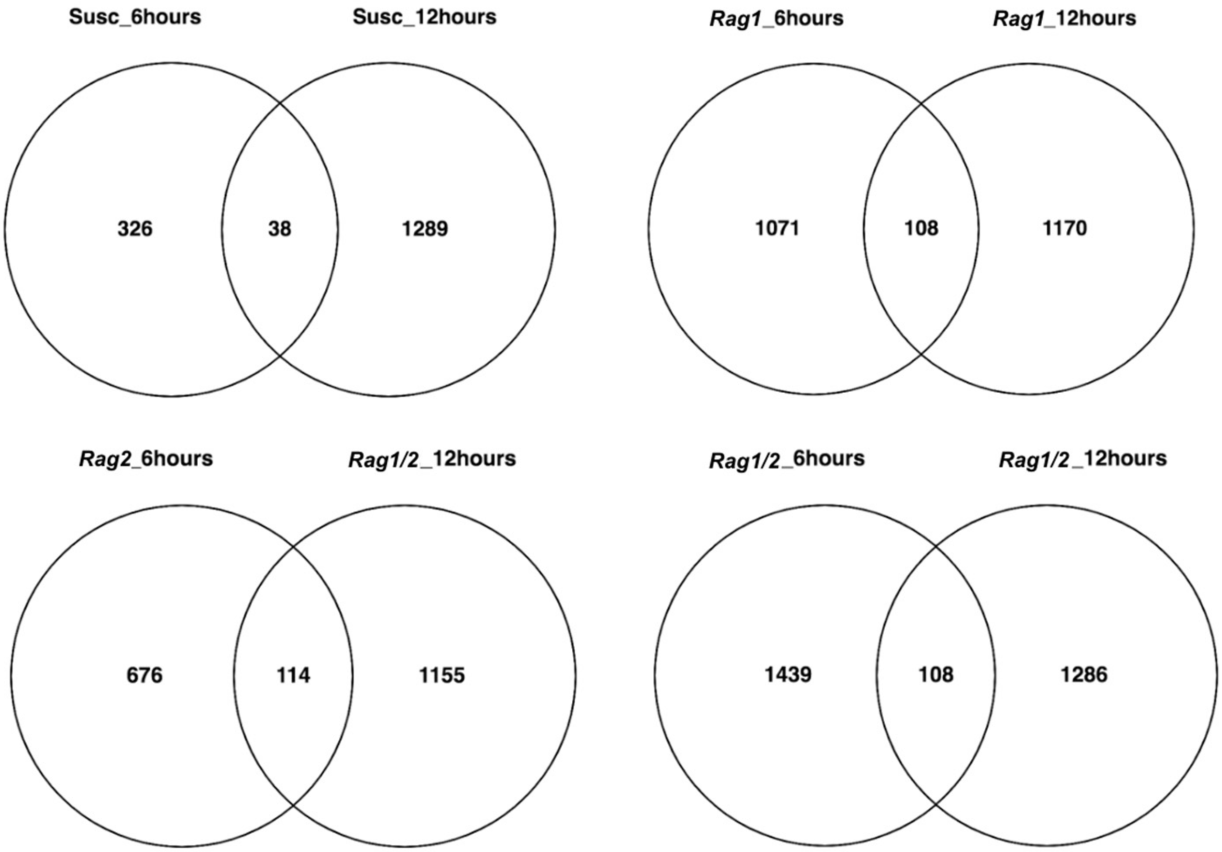

Figure S2. The early (6 h) and late (12 h) responses to aphids are different in all genotypes. Comparison of the transcriptional response of each genotype at different time points.

Additional Figure 3

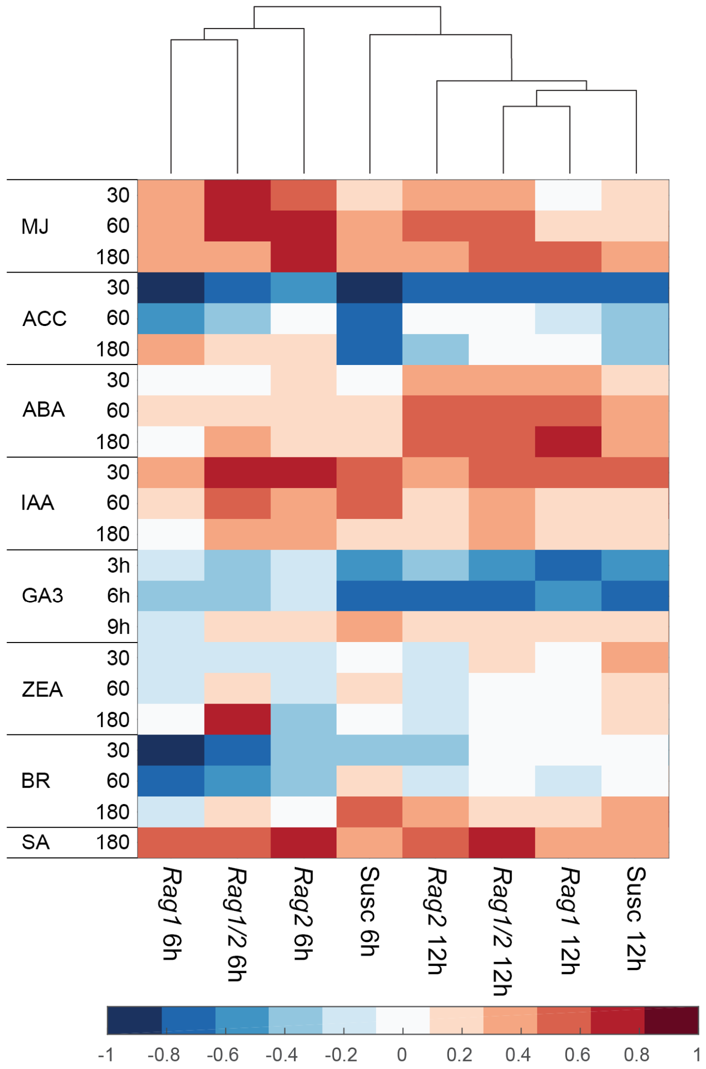

Figure S3. Plant hormone signatures inferred from the transcriptome responses of each genotype. Hormonometer analysis of phytohormone signatures obtained by comparing the response to aphids in each genotype to Arabidopsis datasets of response to different hormones. A correlation index for each genotype/treatment dataset and different hormone treatment datasets, including different hormone treatment times, are indicated. Red colors indicate positive correlation between aphid infestation and a particular hormone response, blue shading indicates negative correlation. MJ, Methyl jasmonate; ACC, 1-aminocyclopropane-1-caroxylic acid (ethylene precursor); ABA, abscisic acid; IAA, indole-3-acetic acid (auxin), GA, GA3 (gibberellin); BR, brassinosteroid; SA, salicylic acid. Numbers next to each hormone indicate different treatment times in minutes unless otherwise indicated.
